# Two common nonsynonymous paraoxonase 1 (*PON1*) gene polymorphisms and brain astrocytoma and meningioma

**DOI:** 10.1186/1471-2377-10-71

**Published:** 2010-08-19

**Authors:** Carmen Martínez, José A Molina, Hortensia Alonso-Navarro, Félix J Jiménez-Jiménez, José AG Agúndez, Elena García-Martín

**Affiliations:** 1 Department of Pharmacology & Psychiatry, Medical School, University of Extremadura, Badajoz, Spain; 2 Service of Neurology, Hospital Doce de Octubre, Madrid, Spain; 3 Department of Medicine-Neurology, Hospital "Príncipe de Asturias", Universidad de Alcalá, Alcalá de Henares, Madrid, Spain; 4 Section of Neurology, Hospital "La Mancha-Centro", Alcázar de San Juan, Ciudad Real, Spain; 5 Department of Biochemistry and Molecular Biology and Genetics, University of Extremadura, Avda de Elvas s/n, 06071, Badajoz, Spain

## Abstract

**Background:**

Human serum paraoxonase 1 (PON1) plays a major role in the metabolism of several organophosphorus compounds. The enzyme is encoded by the polymorphic gene *PON1*, located on chromosome 7q21.3. Aiming to identify genetic variations related to the risk of developing brain tumors, we investigated the putative association between common nonsynonymous *PON1 *polymorphisms and the risk of developing astrocytoma and meningioma.

**Methods:**

Seventy one consecutive patients with brain tumors (43 with astrocytoma grade II/III and 28 with meningioma) with ages ranging 21 to 76 years, and 220 healthy controls subjects were analyzed for the frequency of the nonsynonymous *PON1 *genotypes L55M rs854560 and Q192R rs662. All participants were adult Caucasian individuals recruited in the central area of Spain.

**Results:**

The frequencies of the *PON1 *genotypes and allelic variants of the polymorphisms *PON1 *L55M and *PON1 *Q192R did not differ significantly between patients with astrocytoma and meningioma and controls. The minor allele frequencies were as follows: *PON1 *55L, 0.398, 0.328 and 0.286 for patients with astrocytoma, meningioma and control individuals, respectively; *PON1 *192R, 0.341, 0.362 and 0.302 for patients with astrocytoma, meningioma and control individuals, respectively. Correction for age, gender, or education, made no difference in odds ratios and the *p *values remained non-significant. Haplotype association analyses did not identify any significant association with the risk of developing astrocytoma or meningioma.

**Conclusions:**

Common nonsynonymous *PON1 *polymorphisms are not related with the risk of developing astrocytoma and meningioma.

## Background

Primary cancers of the brain and nervous system globally account for nearly 200,000 new cases per year, the highest rates being observed in developed areas [1]. The two most common histologic types of brain tumors in adults are gliomas and meningiomas, and data suggest that gliomas are more common in men, while meningiomas occur more often in women [2].

The etiology of brain tumors is still poorly understood. Despite some studies suggested a possible relationship between the risk for brain tumors and several occupational and environmental exposures, including farming [3-5] and pesticides and/or herbicides [2,5-10], others failed to show this association [11-13]. A recent multicenter case-control study examining incident glioma and meningioma risk associated with occupational exposure to insecticides and herbicides showed increased risk for meningioma in women who reported ever using pesticides, with a trend of increasing risk with increasing years of herbicide exposure [14]. Interestingly, it has been reported that, in experimental models, organophosphorus insecticides and their oxons can affect astroglial cell proliferation in cultures of astrocytoma-glioma cell lines or primary astrocytes [15,16].

Human serum paraoxonase 1 (PON1), a enzyme encoded by the polymorphic gene *PON1 *on chromosome 7q21.3, is an aryldialkylphosphatase, synthesized mainly in the liver, that plays a major role in the metabolism of several organophosphorus compounds, like some insecticides, neurotoxins, and arylesters [17]. The high variability in the activity of PON1 has been attributed to several polymorphisms within the gene, as well as physiological and pathological states, dietary and lifestyle factors and environmental chemicals. Two nonsynonymous polymorphisms, a leucine to methionine substitution at position 55 (L55M, rs854560, c.220 T > A according to the GenBank accession number NM 000446) and a glutamine to arginine substitution at position 192 (Q192R, rs662, c.632 A > G according to the GenBank accession number NM 000446), 8638 bp apart, have been shown to influence PON1 activity [18-20]. The M allele at position 55 causes a decrease in protein stability [21] and the Q allele at position 192 has been associated with decreased metabolic activity for some substrates [22,23]. In the serum, PON1 is associated with high density lipoprotein (HDL), and plays an important role in lipid metabolism as an antioxidant molecule through several mechanisms [24-26]. In addition, PON1 is implicated in the elimination of carcinogenic lipid-soluble radicals from lipid peroxidation [27].

Although astrocytoma and meningioma arise from completely different types of cells, it cannot be ruled out that some similar features may be involved in their etiology. In some cases meningiomas can mimic astrocytomas and vice-versa and some studies reported concurrent occurrence of both tumors in the same patient [28-35]. Moreover, genetic and non-genetic risk factors have been associated with both types of tumors [36,37]. To establish whether *PON1 *genotype and allelic variants could be related to the risk of developing brain astrocytoma and/or meningioma, we have compared the prevalence of the *PON1*-L55M and *PON1*- Q192R polymorphisms in the *PON1 *gene, in a group of 71 patients with these brain tumors (43 with astrocytoma grade II/III and 28 with meningioma), and 220 healty controls.

## Methods

We studied 43 unrelated patients with brain astrocytoma grade II/III (26 men, 17 women; mean ± SD age 51.7 ± 17.4 years) and 28 with brain meningioma (6 men, 22 women; mean ± SD age 62.1 ± 11.7 years). The age ranges were 21-68 years for astrocytoma and 27-76 years for meningiomas. All consecutive patients attending the participating hospitals (Hospital Universitario "Doce de Octubre" (Madrid, Spain) and the Hospital Universitario Infanta Cristina (Badajoz, Spain)) between 1997 and 1999 that were diagnosed of astrocytoma grade II/III or brain meningioma were included in the study, and none was excluded for any reason. Diagnosis was confirmed by histologic analysis in all patients. These patients participated in a previous study by our group [38]. The control group was composed of 220 healthy unrelated Caucasian Spanish individuals (110 men and 110 women, most of them students or staff from the University of Extremadura and from the participating hospitals). The inclusion criteria were the following: age over 18 and lack of all the exclusion criteria. Exclusion criteria were history of neurological, gastrointestinal, liver or renal disease. The control group had a mean age of 44.5 ± 12.2 years.

All the participants were Caucasian Spanish individuals from the central area of Spain, and were included in the study after giving written informed consent. The protocol was approved by the Ethics Committees of the Hospital Universitario "Doce de Octubre" (Madrid, Spain) and the Hospital Universitario Infanta Cristina (Badajoz, Spain).

A 10 mL venous blood sample was obtained from each individual, collected in EDTA tubes and stored at -80°C until analysis. Genomic DNA was isolated from leukocytes by means of standard procedures. *PON1 *genotyping was carried out by TaqMan assay designed to detect the following SNPs: *PON1 *L55M, rs854560 and Q192R, rs662 (C___2259750_20 and C___2548962_20, respectively, Applied Biosciences Hispania, Alcobendas, Madrid, Spain). The detection was carried out by qPCR in a Eppendorf realplex thermocycler by using fluorescent probes. The amplification conditions were as follows: After a denaturation time of 10 min at 96°C, 45 cycles of 92°C 15 sec 60°C 90 sec were carried out and fluorescence was measured at the end of every cycle and at endpoint. All samples were determined by triplicate and genotypes were assigned both, by the gene identification software (RealPlex 2.0, Eppendorf) and by analysis of the reference cycle number for each fluorescence curve, calculated by the use of CalQPlex algorithm (Eppendorf). For every polymorphism tested, genomic DNA of twenty individuals carrying no mutations, twenty heterozygotes and twenty homozygotes were analyzed, by amplification-restriction as described elsewhere [39-41] and in all cases the genotypes fully corresponded with those detected with fluorescent probes.

The intergroup comparison values and the significance of the gene-dose effect were calculated by using the chi-square test or the Fisher's exact test when appropriate. Logistic regression was performed to verify that age, gender or education did not modify the odds ratios. Statistical analyses were performed using the SPSS 15.0 for Windows (SPSS Inc., Chicago, Illinois, USA). The patient's sample size was determined from allele frequencies observed for healthy individuals with a genetic model analyzing the frequency for carriers of the disease gene. Hardy-Weinberg equilibrium (HWE) was analyzed with the DeFinetti program (http://ihg2.helmholtz-muenchen.de/cgi-bin/hw/hwa1.pl). Haplotype reconstruction was carried out using the program PHASE v2.1.1 with the default model for recombination rate variation [42]. Seven independent runs with 1000 iterations, 500 burn-in iterations and a thinning interval of 1 were performed as described elsewhere [43]. Association tests were carried out with the software package PLINK [44].

## Results

No departures from HWE were observed. The *p *values (Pearson) for HWE departures were as follows: *PON1 *L55M: cases, p = 0.620, controls, 0.142; *PON1 *Q192R: cases, p = 0.962, controls, p = 0.544. The frequencies of *PON1 *genotypes and *PON1 *alleles in patients with brain tumors did not differ significantly from those of controls, both considering astrocytoma plus meningioma cases (Table 1), or astrocytoma and meningioma separately (Table 2). Different genetic models were used to test the genotypic associations. For overall patients the results for association tests for the *PON1 *L55M polymorphism were as follows: Genotypic test, p = 0.879; trend test, p = 0.691, dominant model, p = 0.847, recessive model p = 0.612. Regarding the results for association tests for the *PON1 *Q192R polymorphism in overall patients were: Genotypic test, p = 0.542; trend test, p = 0.282, dominant model, p = 0.279, recessive model p = 0.567. For astrocytoma patients the results for association tests for the *PON1 *L55M polymorphism were as follows: Genotypic test, p = 0.989; trend test, p = 0.886, dominant model, p = 0.884, recessive model p = 0.932. Regarding the *PON1 *Q192R polymorphism, the association test results were: Genotypic test, p = 0.727; trend test, p = 0.465, dominant model, p = 0.424, recessive model p = 0.778. For meningioma patients the results for association tests for the *PON1 *L55M polymorphism were as follows: Genotypic test, p = 0.605; trend test, p = 0.386, dominant model, p = 0.605, recessive model p = 0.323. Regarding the *PON1 *Q192R polymorphism, the association test results were: Genotypic test, p = 0.649; trend test, p = 0.353, dominant model, p = 0.395, recessive model p = 0.524. We tested also the frequencies for haplotypes and the P values for overall patients, astrocytoma patients and meningioma patients, respectively, were as follows: *PON1 *55L+192Q, 0.648, 0.945 and 0.384; *PON1 *55L+192R, 0.568, 0.721 and 0.557; *PON1 *55M+192Q, 0.560, 0.472 and 0.936; *PON1 *55M+192R, 0.291, 0.515 and 0.318.

**Table 1:** *PON1 *genotype and allelic variants of patients with brain tumor (BT) and healthy volunteers.

	BT PATIENTS (N = 73, 146 chromosomes)	CONTROLS (N = 220, 440 chromosomes)	OR (95% CI); P
**GENOTYPES**			

*PON1 *55 Leu/Leu	11 (15.1) [6.9-23.3]	38 (17.3) [12.3-22.3]	0.85 (0.41-1.75); 0.662^1^

*PON1 *55 Leu/Met	32 (43.8) [32.5-55.2]	94 (42.7) [36.2-49.3]	--

*PON1 *55 Met/Met	30 (41.1) [29.8-52.4]	88 (40.0) [33.5-46.5]	1.05 (0.61-1.79); 0.869^2^

*PON1 *192 Gln/Gln	31 (42.5) [31.1-53.8]	109 (49.5) [42.9-56.2]	0.75 (0.44-1.28); 0.295^1^

*PON1 *192 Gln/Arg	33 (45.2) [33.8-56.6]	89 (40.5) [34.0-46.9]	--

*PON1 *192 Arg/Arg	9 (12.3) [4.8-19.9]	22 (10.0) [6.0-14.0]	1.27 (0.57-2.85); 0.576^2^

**ALLELES**			

*PON1 *55 Leu	54 (37.0) [29.2-44.8]	170 (38.6) [34.1-43.2]	0.93 (0.63-1.37); 0.722^3^

*PON1 *55 Met	92 (63.0) [55.2-70.8]	270 (61.4) [56.8-65.9]	--

*PON1 *192 Gln	95 (65.1) [57.3-72.8]	307 (69.8) [65.5-74.1]	--

*PON1 *192 Arg	51 (34.9) [27.2-42.7]	133 (30.2) [25.9-34.5]	1.24 (0.84-1.84); 0.289^3^

The values in each cell represent: number (percentage) and [95% confidence intervals]. ^1^The OR was calculated by using the dominant model. ^2^The OR was calculated by using the recessive model. ^3^The OR was calculated by using the allelic model. Test for trend with the number of variant alleles: *PON1 *55 Leu/Met: H = 0.0957 df = 1 p = 0.7570; *PON1 *192 Gln/Arg: H = 1.1416 df = 1 p = 0.2853.

**Table 2:** *PON1 *genotype and allelic variants of patients with different types of brain tumor.

	Astrocytoma (N = 44, 88 chromosomes)	OR (95% CI); P	Meningioma (N = 29, 58 chromosomes)	OR (95% CI); P
**GENOTYPES**				

*PON1 *55 Leu/Leu	8 (18.2) [6.8-29.6]	1.07 (0.47-2.43); 0.885^1^	3 (10.3) [0-21.4]	0.55 (0.17-1.81); 0.345^1^

*PON1 *55 Leu/Met	19 (43.2) [28.5-57.8]	--	13 (44.8) [26.7-62.9]	--

*PON1 *55 Met/Met	17 (38.6) [24.2-53.0]	0.94 (0.49-1.82); 0.866^2^	13 (44.8) [26.7-62.9]	1.22 (0.57-2.62); 0.619^2^

*PON1 *192 Gln/Gln	19 (43.2) [28.5-57.8]	0.77 (0.41-1.48); 0.442^1^	12 (41.4) [23.5-59.3]	0.72 (0.33-1.56); 0.409^1^

*PON1 *192 Gln/Arg	20 (45.5) [30.7-60.2]	--	13 (44.8) [26.7-62.9]	--

*PON1 *192 Arg/Arg	5 (11.4) [2.0-20.7]	1.15 (0.43-3.14); 0.786^2^	4 (13.8) [1.2-26.3]	1.44 (0.48-4.34); 0.531^2^

**ALLELES**				

*PON1 *55 Leu	35 (39.8) [29.5-50.0]	1.05 (0.66-1.67); 0.842^3^	19 (32.8) [20.7-44.8]	0.77 (0.44-1.38); 0.386^3^

*PON1 *55 Met	53 (60.2) [50.0-70.5]	--	39 (67.2) [55.2-79.3]	--

*PON1 *192 Gln	58 (65.9) [56.0-75.8]	--	37 (63.8) [51.4-76.2]	--

*PON1 *192 Arg	30 (34.1) [24.2-44.0]	1.19 (0.74-1.94); 0.474^3^	21 (36.2) [23.8-48.6]	1.31 (0.74-2.31); 0.355^3^

The values in each cell represent: number (percentage) and [95% confidence intervals]. ^1^The OR was calculated by using the dominant model. ^2^The OR was calculated by using the recessive model. ^3^The OR was calculated by using the allelic model. Test for trend with the number of variant alleles: Astrocytoma *PON1 *55 Leu/Met: H = 0.0360 df = 1 p = 0.8496; *PON1 *192 Gln/Arg: H = 0.5537 df = 1 p = 0.4568. Meningioma PON1 55 Leu/Met: H = 0.5987 df = 1 p = 0.4391; PON1 192 Gln/Arg: H = 0.8057 df = 1 p = 0.3694.

Correction for age, gender, or education, made no difference in odds ratios and the p values remained non-significant.

## Discussion

The brain is partially protected from chemical insults by a physical barrier mainly formed by the cerebral microvasculature, which prevents penetration of hydrophilic molecules into the cerebral extracellular space [45]. However, several drugs and environmental pollutants, including organophosphorus insecticides or other xenobiotics could reach the brain. This organ possesses an enzymatic equipment able to metabolize xenobiotics, like an entirely functional cytochrome P450 mono-oxygenase system in rodents and humans that would metabolise xenobiotics resulting in the formation of reactive and toxic metabolites in the neuronal cells [46]. Present mainly in the liver and blood, PON1 should hypothetically act as a detoxifying enzyme at this level, causing the hydrolysis of the acetylcholinesterase-inhibiting oxons (activated intermediates) of some organophosphorus compounds [47,48], decreasing the possible arrive of these compounds to the brain. Several evidences point to pesticides as risk a factor for brain tumors [49,50]. PON1 plays a prominent role among the enzymes that prevent or mitigate damage caused by reactive oxygen species. And hence it is conceivable that changes in PON1 activity due to nonsynonymous polymorphisms may modulate the risk to develop brain tumors.

Published evidences make it difficult to determine *a priori *which PON1 isoform represents a risk factor for the development of brain tumors. Although initial findings point to the *PON1*-55M and *PON1*-192Q [21-23], it should be remarked that there exists a differential activity of *PON1*-192Q genotype towards different substrates [51]. For that reason, besides genotypes, we analyzed all possible haplotypes and diplotype combinations as putative risk factors, and we explored all genetic association models. Our findings did not indicate association of the risk either with genotypes, allele frequencies, haplotypes or diplotypes.

A few previous reports addressed the possible role of *PON1 *polymorphisms in the risk for brain tumors: Searles-Nielsen et al. observed no main effects or interactions with insecticides for the Q192R and/or L55M SNPs, but suggested that the functional C-108T polymorphism and insecticide exposures may be important [52,53] Kafadar et al. [54] studied *PON1-Q192R *polymorphism and serum PON1 activity in 42 patients with high grade gliomas, 42 patients with meningioma, and 50 controls. Although they found in both tumor groups decreased PON1 activity when compared with controls, *PON1-Q192R *genotype and allelic variants did not differ between the study groups. Rajamaran et al. [55] studied diverse gene polymorphisms related to oxidative response, including the *PON1-Q192R *polymorphism, in patients with glioma, meningioma, and acoustic neuroma. No association of the *PON1-Q192R *polymorphism with the risk of developing any of these tumors was identified.

In the present study we found no significant differences either in *PON1-*55 or *PON1-*192 allele frequencies or genotype frequencies between patients with meningioma or grade II/III astrocytoma, as compared with healthy control subjects.

A limitation of this study is that control subjects are younger than patients and that it cannot be ruled out that some control individuals would eventually develop brain tumors. Nevertheless, the possibility that some healthy subject would eventually develop these tumors in the lapse between the mean age of controls and the mean age of cases is negligible given the prevalence of these tumors in the studied population, and therefore the differences in the mean age of patients and controls reflects that the control group is not fully comparable to cases, but it should not influence the findings obtained in the present study. Regarding the geographical origin of the patients and controls, no genetic differences are expected because all participants were Spanish Caucasians living in close areas and because in previous genetic studies we have not detected any genetic differences between individuals from Extremadura and Madrid [56-60]. Another limitation of this study is the absence of data regarding exposure to chlorpyrifos or diazinon. Nevertheless, it should be stated that a recent study that identified interaction between exposure to insecticide treatment and some polymorphisms of pesticide metabolism genes failed to identify a significant interaction of exposure with the nonsynonymous *PON1 *polymorphisms analyzed in the present study [53]. Additional limitations are the inability to analyze other functional *PON1 *SNPs, such as the highly functional C-108T SNP, and the lack of PON1 activity measurements, although this does not invalidate the findings indicating the lack of a major genetic association with the SNPs analyzed in this study. In fact, clinical association of *PON1 *polymorphisms, but not PON1 enzyme activity, with ischemic stroke has been recently demonstrated [61] and vice-versa, no association between adult brain tumors and *PON1 *genotype, but positive association with PON1 activity has been described [54]. In this regard, Furlong *et al. *recommend both, genotype determination and measurement of serum enzyme activity for evaluation of PON1's role in risk of disease or exposure [62].	Another limitation of this study is the sample size of subgroups of patients according to the histological type of tumor. In this study we cannot exclude a false negative result due to the sample size. Nevertheless, the study is sufficiently powered to rule out a major association of *PON1 *polymorphisms. For patients with astrocytoma the study can rule out an association with OR ≥ 2.1, and for patients with meningioma the study can rule out an association with OR ≥ 2.5. Sporadic disease-genotype associations this strong are extremely rare, particularly with cancer risk [63-65]. Even considering this limitation, this study indicates the absence of a major association of the nonsynonymous *PON1 *polymorphisms studied with brain tumors.

## Conclusions

Common nonsynonymous *PON1 *polymorphisms are not related with the risk of developing astrocytoma and meningioma.

## Competing interests

The authors declare that they have no competing interests.

## Authors' contributions

CM: acquisition of the data, critical revision, obtaining funding; administrative, technical and material support. JAM: acquisition of data; critical revision. HAN: conception and design, acquisition of the data, analysis and interpretation of the data, drafting of the submitted material, critical revision, administrative, technical and material support. FJJJ: conception and design, analysis and interpretation of the data, drafting of the submitted material, critical revision, administrative, technical and material support, and supervision. JAGA: conception and design, analysis and interpretation of the data, drafting of the submitted material, critical revision, statistical expertise, obtaining funding, and supervision. EGM: conception and design, analysis and interpretation of the data, drafting of the submitted material, critical revision, obtaining funding, and supervision.

All authors read and approved the final manuscript.

## Pre-publication history

The pre-publication history for this paper can be accessed here:

http://www.biomedcentral.com/1471-2377/10/71/prepub
